# Graduate Entry Medicine: Selection Criteria and Student Performance

**DOI:** 10.1371/journal.pone.0027161

**Published:** 2011-11-21

**Authors:** Owen Bodger, Aidan Byrne, Philip A. Evans, Sarah Rees, Gwen Jones, Claire Cowell, Mike B. Gravenor, Rhys Williams

**Affiliations:** School of Medicine, Swansea University, Swansea, United Kingdom; University of Illinois at Champaign-Urbana, United States of America

## Abstract

**Background:**

Graduate entry medicine raises new questions about the suitability of students with different backgrounds. We examine this, and the broader issue of effectiveness of selection and assessment procedures.

**Methods:**

The data included background characteristics, academic record, interview score and performance in pre-clinical modular assessment for two years intake of graduate entry medical students. Exploratory factor analysis is a powerful method for reducing a large number of measures to a smaller group of underlying factors. It was used here to identify patterns within and between the selection and performance data.

**Principal Findings:**

Basic background characteristics were of little importance in predicting exam success. However, easily interpreted components were detected within variables comprising the ‘selection’ and ‘assessment’ criteria. Three selection components were identified (‘Academic’, ‘GAMSAT’, ‘Interview’) and four assessment components (‘General Exam’, ‘Oncology’, ‘OSCE’, ‘Family Case Study’). There was a striking lack of relationships between most selection and performance factors. Only ‘General Exam’ and ‘Academic’ showed a correlation (Pearson's r = 0.55, p<0.001).

**Conclusions:**

This study raises questions about methods of student selection and their effectiveness in predicting performance and assessing suitability for a medical career. Admissions tests and most exams only confirmed previous academic achievement, while interview scores were not correlated with any consequent assessment.

## Introduction

Graduate Entry Medicine (GEM) is a relatively new concept to UK medical schools which have traditionally recruited medical students predominantly straight from secondary education [1]. Swansea University's School of Medicine has recently enrolled its seventh cohort of students to its GEM programme. The first group (enrolled in 2004) graduated at the end of the academic year 2007/2008. Swansea GEM students come from a diverse a range of backgrounds, having studied a variety of first degree subjects, ranging from arts and humanities to pure science, posing a considerable challenge for those responsible for course design and the admissions process.

Although our study group are exclusively high-achieving, and therefore of reduced variance in any measure of performance when compared to the population as a whole, there is a growing body of literature on the extent to which graduate entry medical student achievement and the student experience are measurably influenced by students' characteristics and background, including previous tertiary education. For example, a study from Newcastle, New South Wales [2] examined the results of the first assessment and final assessment in the first year of 303 “standard” scheme students admitted to the course from 1990 to 1998. They concluded that: “there are some medical student groups who encounter more academic difficulties than others in the first year” and went on to suggest that “identifying these students can assist medical schools to focus academic support appropriately”. Another study [3] also found that background mattered, concluding that students with science based first degrees did better in certain aspects of the course, although the differences were small and diminished as the course progressed. The importance of admissions tests for medical school applicants has also been investigated. The issue of specific aptitude tests was examined by Mitchell [4] in the United States. They found that the US Medical College Admissions Test (MCAT) predicted grades only slightly better than performance at school. In a more recent study [5] no relationship was found between performance in the Graduate Australian Medical School Admission Test (GAMSAT) and either the development in clinical reasoning ability or interview score. Interview scores did, however, prove to a useful indicator of ability. No such link was identified by Rahbar [6], whose findings suggested that interview ratings were not related to scholastic performance. These findings were broadly borne out in research carried out in the University of Queensland [7], where they concluded that “GAMSAT is poor at predicting academic performance” while interview offered “weak to modest” predictive performance.

The UK currently has 14 locations offering medical courses specifically directed towards graduate students. Some, such as Birmingham University, stipulate that first degrees must be in a life science discipline. Others, including Swansea, accept applications from any discipline. Swansea's current policy is to recruit graduates with first degrees with a 1st or upper second class classification. Given the availability of performance data on two full cohorts of students, up to and including the 2^nd^ MB examinations, we decided to investigate this in relation to data used to inform the selection process, particularly those relating to the previous academic careers, as selection of the best students is likely to be crucial in producing high quality doctors. Given the large number of variables collected both on the student background and on the assessment during examination, a straightforward statistical analysis faces two problems. The first is that due to multiple testing of a large number of comparisons, there is a high risk of false positive correlations. Secondly, due to the expected correlation between many of the measures, it is difficult to test for combined effects of the measures. Here we take a multivariate statistical approach, which allows us to first characterise the complex student selection and assessment data sources into a reduced number of underlying key factors, and second to test for links between the resulting selection and performance indicators.

## Methods

### Data source

The GEM programme leading to the University of Wales MBBCh degree is a four-year course administered, for the cohorts included in this study, by Swansea University for its first two years and by Cardiff University for its two concluding years. The target for recruitment is now 70 students per year, however the first cohort consisted of half this number of students. In this study we have analysed the in-course assessment results up to the end of the second year for the first two cohorts of students (i.e. those entering the programme in 2004/2005 and 2005/2006). This two-year set of data comprise all assessment modules undertaken during the pre-clinical phase of teaching (up to an including 2^nd^ MB examinations).

Access to the data was as part of a routine quality improvement exercise conducted annually on exam results, with the aim of improving the course. Anonymous data was used at all times. For these reasons we did not feel it was necessary either to seek permissions from the students or to obtain ethical approval to use this routinely collected data.

### Data structure

We define three distinct classes of data: demographic, prior academic record and assessment. The first two classes of data were available during the selection process, while assessment data consists of marks awarded to the student for performance in elements of the taught course. A full list of variables included in the analysis is given in Table 1, with each identified as either a continuous covariate or as a categorical variable (with only a small number of discrete categories). The number of categories used has been kept as small as possible, due to sample size restrictions, and full details are given in Table 2. Although some of these variables are of nominal type, and others ordinal in nature we have collectively referred to all discrete variables of low order as categorical. First degree subject classifications are the same as those adopted by De Clercq [2]. All variables are used in the analysis with the exception of the full details of A-level qualifications. Instead we concentrate only on results in Maths and English.

**Table 1:** Summary of variables of the data set included in the analysis.

Group	Variable
Demographic:	Gender (f)
	Year of intake (f)
	Nationality (f)
	Ethnicity (f)
	Age (c,f)
	Years since completion of most recent period of study (c)
Selection	Points at GCSE (c)
	GCSE Maths, English, Science (f)
	A level subjects & grades (n,f)
	A level Maths, English (f)
	Points at A-level (c)
	First Degree Subject Area (f)
	First Degree Grade (f)
	Higher degree (f)
	Degree University (f)
	Interview Scores (c)
	GAMSAT UK (c)
Assessment (Modules)	Alimentary (c)
	Cardiovascular & Respiratory (c)
	Development, Growth & Reproduction (c)
	Health in Society (c)
	Homeostasis (c)
	Human Structure (c)
	Infection and Immunity (c)
	Mechanisms of Disease & Therapeutics (c)
	Musculoskeletal (c)
	Neuroscience (c)
	Family Case Study (c)
	Oncology Case Study (c)
	Objective Structured Clinical Exam (OSCE) (c)

Footnote: (f) denotes a categorical variable, (c) a continuous covariate and (n) a nominal variable.

**Table 2:** The coding categories for categorical variables.

Factor	Categories
Age band	21 to 23, 24 to 26, 27 or over
Gender	Male, Female
Year of intake	2004, 2005
Nationality	British, Other
Ethnicity	White, Other
GCSE Maths, English	A or A*, B or C or D for each subject
GCSE Science	1,2 or 3 subjects taken
A level subjects & grades	Subject names and grades (A, B, C)
A level Maths, English	Yes, No for each subject
First Degree Subject Area	Health Professions, Biomedical Science, Other Biology, Physical Sciences, Non-Science.
First Degree Grade	1^st^, 2∶1, 2∶2
Higher degree	Yes, No
Degree University	English, Welsh, London, Oxford/Cambridge

### Selection measures

The selection measures are all drawn from the information submitted by the student in their initial application. The variables available to us represent three different sources: prior school performance, GAMSAT scores and the outcome of the interview process. The Graduate Medical School Admissions Test (GAMSAT) UK is a series of three examination papers to assess problem-solving, data interpretation, critical thinking, reasoning and written communication [8]. Each of the papers has a different emphasis, with the first paper focussing on reasoning in humanities and social science, the third paper focussing on reasoning in biological and physical sciences and the second paper testing written communication skills.

The interview phase consisted of one meeting with two trained, senior teaching staff, with a mean taken of their two scores, each of which was based equally on performance in six domains (i.e. motivation and interest in studying medicine; understanding the demands of medical training; caring ethos & sense of social awareness; sense of responsibility; evidence of a balanced approach to life; ability to communicate effectively/interpersonal skills).

### Assessment measures

Each of these measures represents the mark awarded for one of the modules taken during the first two years of the course. All modules undertaken during the pre-clinical phase of teaching are included. Details of the modules are given in Table 3 and Table 4. A mark is a combination of the outcome of examinations and any assessed coursework submitted. Where modules ran for two years the method of assessment was the same for both years and there was almost always a very significant correlation between the first and second year marks (p<0.01). As a consequence where modules span both years the marks have been combined (with equal weighting) into one average mark. The one exception to this was Infection and Immunity, which showed no significant correlation (p = 0.339). No satisfactory explanation for this could be found beyond an observation made by staff involved in assessment that it was common for one module each year to produce atypical results. In this case it appears that the results from the first year Infection and Immunity module for the 2005 intake were sufficiently at odds with other results to nullify the correlation between first and second year marks. Three modules do not run for two years i.e. Human Structure, Family Case Study and Oncology Case Study. The Objective Structured Clinical Examination (OSCE) takes the form of a practical assessment held only at the end of the second year.

**Table 3:** A summary of the modules studied during the pre-clinical phase of teaching.

	Years	Assessment	Inter-year Correlation
Alimentary	1,2	Exam, Coursework	0.41**
Cardiovascular & Respiratory	1,2	Exam, Coursework	0.38**
Development, Growth & Reproduction	1,2	Exam, Coursework	0.45**
Health In Society	1,2	Exam, Coursework	0.33**
Homeostasis	1,2	Exam, Coursework	0.53**
Human Structure	1	Examinations (3)	-
Infection and Immunity	1,2	Exam, Coursework	0.09
Mechanisms of Disease & Therapeutics	1,2	Exam, Coursework	0.58**
Musculoskeletal	1,2	Exam, Coursework	0.38**
Neuroscience	1,2	Exam, Coursework	0.41**
Family Case Study	1	Coursework	-
Oncology Case Study	2	Coursework	-
OSCE Exam	2	Practical Exam	-

Footnote: Correlation significant at the 1% level is indicated by **.

**Table 4:** Brief descriptions of the content of modules studied in the pre-clinical phase of teaching.

Module Descriptions
Alimentary: establishes the fundamentals of the anatomy and physiology of the normal gastrointestinal tract and considers the clinical presentation of a variety of disorders.
Cardiovascular & Respiratory: covers the physiology of the heart, circulatory and respiratory systems. It introduces clinical and public health aspects of cardiorespiratory disease, and common respiratory conditions.
Development, Growth & Reproduction: covers the normal physiology of conception, pregnancy and childbirth, that aging is a normal process with structural, physiological and psychological consequences, and introduces clinical genetics and the effects of maturation.
Health in Society: covers basic principles of health psychology, social factors, illness at different phases of life, ethical/legal issues and critical appraisal of published evidence. It covers epidemiology and statistical approaches, Public Health and preventative medicine.
Homeostasis: focuses on the endocrine system (abnormalities, detection and management), the kidney and the acid-base balance and metabolism.
Human Structure: considers the human anatomy important to clinical medicine. Topics include the back, head and neck and the locomotor, cardiovascular, respiratory, digestive and urogenital systems with emphasis on practical classes.
Infection & Immunity: provides an introduction to pathogenic organisms, infection, and the mechanisms of resistance through immunity. It explores immune response, immunity problems, the clinical approach to infection and anti-microbial drugs
Mechanisms of Disease & Therapeutic Approaches: focuses on mechanisms leading to disease and detection, diagnosis and treatment of disease emphasising drug therapy. It explores the genesis and progress of disease and how effective therapeutic strategies can be implemented.
Musculoskeletal: consolidates anatomical knowledge of the limbs and back including the biochemistry and physiology of connective tissue, bone and muscle. Disorders of the musculoskeletal system, including inflammatory joint disease, repetitive motion disorders, common muscle injuries and fractures are covered.
Neuroscience: focusses on the nervous system, and the consequences of injury or disease. Included are an introduction to history-taking and the relevant physical examination, the clinical diagnostic process and the treatment and care of patients with neurological disease.
Family Case Study: develops insight into an important area of medical practice and interdisciplinary care - the birth of a child. Students will be attached to a family, with a new baby, and will write a reflective, referenced report linked to guidance from the GMC document Good Medical Practice 2006.
Oncology Case Study: develops insight into an important area of medical practice and interdisciplinary care - the personal impact of cancer. Students will be attached to care workers involved with a patient with cancer, and will write a reflective report on their experiences.
OSCE: is an exam based around a circuit of short stations, in which each candidate is examined on a one-to-one basis with either real or simulated patients. The mastery of clinical skills is tested rather than pure theoretical knowledge.

### Statistical methods

The intention of this analysis is twofold. First, we wanted to explore the selection and assessment data to understand the extent to which the different measures provide original information, allowing us to identify the different, unique, dimensions within each set. Each measure may represent a different aspect of a student's record or cover a unique part of the syllabus, with some having unique methods of assessment, but there was a suspicion that many of the indicators were highly correlated and may provide relatively little original information about a candidate's suitability or prospects. For example, it is not obvious whether combined aggregate measures of academic performance (such as points at A-level) will correlate well with marks awarded during selection interview, or during a practical assessment. The second main motivation of this study was to explore the extent to which the selection measures can be seen to predict the different facets of a student's aptitude.

The analysis has been split into three parts, all of which were conducted in SPSS v13. The first part dealt with the simple univariate relationship between individual selection variables and a student's overall performance. We sought to address questions such as whether the first degree subject has any influence on performance and for this we used t-test and ANOVA approaches. Those variables that showed little correlation with any aspect of assessment were dropped from the analysis at this stage.

The second part of the analysis aimed to take into account the correlations between many of the measures and to simplify the data set by identifying underlying factors shared by the different measures. At this stage we still had quite a large number of highly correlated continuously distributed variables. The area of Exploratory Factor Analysis incorporates Principal Component Analysis (PCA), which is a well established approach for identifying patterns in, and reducing the complexity of, such datasets without imposing too many of ones preconceptions on the outcome. We initially applied PCA separately to the Selection and Assessment measures, reducing both sets of measures to a small set of key components. Using two PCAs in this way has an advantage over canonical correlation analysis in that it will model all dimensions of the dataset, regardless of their potential for predicting performance. Many multivariate methods are best suited for use with data exhibiting multivariate normality (MVN), and despite most of our variables passing univariate tests for Normality, the data as a whole fails the (stringent) Shapiro-Wilk test for MVN. PCA itself makes no distributional assumptions when used in a descriptive capacity [9], and given that our study is exploratory and we do not wish to test any specific hypotheses regarding the factor structure of the dataset we felt that it was a suitable tool to use. We then transformed each of our continuous variables onto a 5-point ordinal scale (based on quartiles) and performed the non-parametric categorical PCA (also in SPSS) to ensure our findings were robust.

In the third part of the analysis a comparison was made to see whether there was any correlation between the identified main components of ‘selection’ and those of ‘performance’. We therefore aimed to first identify the main characteristics of the complex Selection and Assessment data sets, and second to identify any strong links between them. This was done by examining the correlation between variables drawn from the two sets. As we were concerned that a reliable test of significance was not possible when using factor models build using data that is not MVN, we compared our findings against the results of a non-linear canonical correlation.

## Results

### Participants

The data consisted of 105 records, with 37 and 68 from the 2004 and 2005 intakes respectively. 3 students had to be excluded from the full set of student records on the grounds of incomplete data due to non-completion of the course. The sample size is smaller than many of the previous studies, which range from 189 [10] to over 300 [2], [3]. However, many of the effect sizes observed in these studies were sufficiently large to be detected if present in our study. We will consider the impact of the sample size in more detail when discussing our results.

### Overall performance

The first stage of analysis was to examine, separately, the influence of background variables on the final mark, which is an average of marks in all modules studied during the pre-clinical phase of teaching. A summary of results are given in Table 5 and Table 6. Relatively little of interest was found among the categorical variables with some showing a weak relationship with the final grade but rarely at a significant level. Educational background had little relationship to the final mark, neither the subjects studied at A-level nor the subject of the undergraduate degree. Even the final degree class was not significant as a predictor (p = 0.065), its effect size having a 95% confidence interval of −0.1 to 4.0 in favour of those with first class degrees. When students were classified into groups dictated by their initial degree subject, there were neither significant nor consistently large effects observed to allow us to draw any firm conclusions. It is possible that a much greater sample size would give the test greater statistical power and significant results may become apparent, but effect sizes were not observed to be large. It should be noted that, where such a comparison was possible, we observed much smaller effect sizes in general than other researchers [10].

**Table 5:** Impact of categorical background variables on performance.

Category	Categorical Variable	First Year Mark (p-value)	Final Mark (p-value)	Impact on Individual Module Marks where significant (p-value)
Demographic	Age band	0.279	0.039	None
	Sex	0.375	0.540	None
	Ethnicity	0.229	0.778	None
	Nationality	0.434	0.978	None
	Intake	0.001	0.161	Homeostasis (p<0.001), Neuroscience (p<0.001)
Selection	GCSE Maths	0.447	0.517	None
	GCSE English	0.245	0.084	None
	A level Maths	0.100	0.074	None
	A level English	0.559	0.227	None
	Degree Subject	0.622	0.936	None
	Degree Class	0.014	0.065	Human Structure (p<0.001)
	Higher degree	0.652	0.249	None
	Degree University	0.228	0.080	None

**Table 6:** Impact of continuous covariates on performance.

Continuous Covariate	Final Year Mark (p-value)	Effect Direction	Effect Size (in percentage points)
Age	0.645	-	-
Years since most recent study	0.062	-	-
GCSE points	<0.001	Positive	1% per 50 points
A level points	<0.001	Positive	1% per 2.5 points
Interview Score	0.082	-	-
GAMSAT	<0.001	Positive	1% per 4.3 marks

All of the continuous covariates showed significant correlation levels (p<0.001), with the exception of Age. It has been observed [10] that measures of reasoning ability are less useful than measures of knowledge as indicators of academic performance or clinical ability. Our findings provided some support for this, although the effect sizes were very small. As a predictor for the final mark we found A-level points to be the most useful (p<0.001 with an increase in 50 A-level points corresponding to a predicted 1% increase in Final Mark), followed by GCSE points (p<0.001 with a 2.5 point gain predicting a 1% increase) and GAMSAT score (p<0.001, a 4.3 mark gain predicting a 1% increase). Age provided a mixed picture, with broad age bands being significant (p = 0.039) under ANOVA but no simple linear trend in the relationship (the youngest and oldest students tending to perform slightly better than those in between).

### First year performance

Although we found in the first part of this analysis that previous study did not appear to show a strong impact on the final mark it is possible that relevant previous study or experience will confer a short term benefit to some students. Craig [3] concluded that the previous degree could give a small advantage in certain subjects but that this quickly diminished as the course progressed.

In order to explore this possibility we considered each of the categorical variables against the overall mark for the first year. We found that when that data was split by intake, the 2004 group outperformed the 2005 group significantly (p = 0.001). Although there was some evidence that final degree mark was also influential it was below the level of significance. The only evidence found for an initial advantage based on prior education was when considering the effect on individual module marks.

A level Maths seemed to be associated with better performance in several modules, but this disappeared once corrections were made for multiple testing using the Holm-Bonferroni method. A level English seemed to be associated with improved performance in the Family Case Study but this also fell below the threshold for significance under multiple testing. The only relationships to survive this correction were a significantly better performance in Homeostasis and Neuroscience (p<0.001 for both) by the first cohort over the second and a better mark in Human Structure (p<0.001) for those with a first class degree. We had already identified the continuous covariates as being associated with higher overall marks and so no comparison was made between them and the first year marks.

### Impact of background variables and sample size

Overall we found that the background of students made little systematic difference to their performance during assessment. This contrasted with previous research that showed a link between previous work or study and performance, particularly in the first year. As each of these papers used a very different method for assessing performance a simple comparison with our study was not always easily made.

In [10] a comparison was made of mean marks between different categories of student. They observed males students, under 25's and those who had studied the biological sciences as scoring 0.47, 1.2 and 3.72 marks higher. When examining the same groups we found disparities of −0.58, 0.08 and 0.03 respectively. Given the standard deviation of the final year marks, if our sample was split into two equal groups the threshold for significant difference would be around 1.7.

In [2] assessment is made over five domains: evaluation of professional skills, critical reasoning, identification prevention and management of illness, population medicine and self directed learning. Students are judged as satisfactory or non-satisfactory in any one or more domains. Comparisons were made between different groups of students by evaluating the relative risk of falling into the unsatisfactory category. In order to attempt a comparison we took the lowest 21% of first year marks to represent the 21% of De Clercq's students who were unsatisfactory. They noted relative risks for non-science students, those with 2^nd^ class degrees and females of 3.9, 2.9 and 1.8 respectively. We observed relative risks of 1.13, 3.94 and 0.78 for these same categories. In the case of non-science and female students the relative risk we observed was significantly lower than De Clercq, and not significantly different from 1. For our sample a relative risk of around 2.6 would mark the threshold of significance.

In almost all cases we find that we observe much smaller differences between the groups than in these other papers. Not only this, but many of their results would have also been significant had they been observed in our study, despite the smaller sample size. In summary our findings support those of Craig [3] in “finding little or no difference in performance by medical students from various academic backgrounds”, irrespective of the small sample size.

### Multivariate factor analysis of patterns in the selection criteria

The factor analysis aims to identify a reduced number of underlying characteristics (factors) that explain the full set of measures. Of the full set of variables available from the students' previous records and considered in the initial analysis, a screen was performed to eliminate those that were either very poor indicators of performance or very strongly correlated with other measurements. This restricted our final analysis to 7 variables: GCSE points, A-level points, GAMSAT papers 1, 2 and 3, Interview Score and Age. Although age appeared somewhat out of place with the other performance related variables it did show a relationship with final marks in the initial analysis and we found it changed the results very little, correlating neatly with the resulting second factor.

We considered several tests of factorability. The Kaiser-Meyer-Olkin measure of sampling adequacy was 0.694, well above the recommended value of 0.5 and the result of Bartlett's Test of Sphericity was significant (χ^2^(21) = 100.3, p<0.001). Both of these tests indicate that the partial correlations between variables are not small, and so factor analysis is an appropriate tool. Principal components analysis was used as our starting point from which we explored various rotations. The initial eigenvalues showed that the first three factors explained (cumulatively) 34%, 55% and 67% of the variance respectively. A Quartimax rotation was then performed as it proved to be the best way to separate the variables into clear factor groups.

The three-factor solution could account for 67% of the total variation and was preferred over the two factor solution as it resulted in much higher communalities. The two factor model had three factors with communality below 0.5, but three factors gave us a minimum communality of 0.576. The factor loading matrix for the rotated solution is given in Table 7. In this form the factors appeared to have quite clear interpretations. We found that Factor 1 (which we term ‘Academic Record’) seemed to represent a student's previous academic record. Factor 2 (‘GAMSAT Ability’) seemed to represent average performance in GAMSAT 2 and was also correlated with the GAMSAT 1 and 3 exams (which are also correlated with the Academic Record factor). The performance in interviews was not correlated with anything else in the whole dataset (neither selection nor assessment variables) and ended up in a category of its own, namely ‘Interview Ability’ (Factor 3). Thus the full set of selection information can be boiled down to three separate and *uncorrelated* underlying factors that capture the majority of the information: ‘Academic Record’, ‘GAMSAT’ and ‘Interview’.

**Table 7:** The loading matrix for the factor analysis of measures available during the selection process.

	Factor 1: ‘Academic Record’	Factor 2: ‘GAMSAT Ability’	Factor 3: ‘Interview Ability’	Communality
GCSE points	.81			0.70
A-level points	.78			0.62
GAMSAT 1	.58	.55		0.64
GAMSAT 2		.75		0.58
GAMSAT 3	.59	.43		0.58
Sum of Interview Marks			.95	0.93
Age		.76		0.62

Footnote: Note that factor loadings <0.3 are suppressed.

An almost identical factor model was returned by the non-parametric approach, supporting the robustness of our findings. The only slight deviation from the standard PCA was that Age was much more strongly associated with ‘GAMSAT Ability’ and GAMSAT 1 showed a weak correlation with ‘Interview Ability’.

### Multivariate factor analysis of patterns in the assessment criteria

Choosing the assessment variables to be included was a much simpler task. The variables consisted mostly of percentage scores for different modules studied during the two-year taught course. The module titles and characteristics are given in Table 3. Tests of suitability for factor analysis of the assessment variables also received results with clear, positive, interpretations. The Kaiser-Meyer-Olkin measure of sampling adequacy was 0.906 while Bartlett's test of Sphericity was significant (χ^2^(78) = 745.4, p<0.001). We applied the same method as before (principal components analysis) and found that the same rotation (Quartimax) provided the best results. The initial eigenvalues showed that the first four factors explained (cumulatively) 50%, 61%, 68% and 75% of variance respectively. The ‘eigenvalue greater than 1’ rule proposed a two-factor model, but this reflected the fact that several of our variables seemed to be independent of each other and the rest of the data. In order not to exclude these we opted for the four-factor model, which allowed our model to describe all of the variables effectively. This can be seen in the communality values, which were all greater than 0.5.

The factor loading matrix for the rotated solution is given in Table 8. Again we find quite clear ‘meanings’ for the factors. All of the modules that were tested by examination were highly correlated with each other. Given that examination is the dominant form of assessment we termed this factor ‘General Ability’. To avoid confusion with the *selection factors* 1 to 3, described above, we refer to this as Factor 4. The remaining factors represented the performance in key modules that did not depend so heavily on examination. Factor 5, ‘Oncology’, was highly correlated with the Oncology mark, and to a lesser extent with Health in Society and Infection and Immunity. Factor 6 (OSCE) presented a similar picture, well correlated with the OSCE mark and with other minor correlations. The final factor, ‘Family Case Study’ (Factor 7) was unrelated to anything other than the Family Case Study mark. As before, we obtained a robust factor solution. This was not entirely unexpected given the very high correlations between most of the module marks. Thus the analysis again identified a clear structure in the data.

**Table 8:** The loading matrix for the factor analysis of Assessment measures.

	Factor 4: ‘General Ability’	Factor 5: ‘Oncology’	Factor 6: ‘OSCE’	Factor 7: ‘Family Case Study’	Communality
Alimentary	.81				0.67
Cardiovascular & Respiratory	.79				0.64
Development, Growth & Reproduction	.84				0.75
Health in Society	.78	−.31			0.77
Homeostasis	.83				0.72
Human Structure	.70		.42		0.71
Infection and Immunity	.57	.44			0.59
Mechanisms of Disease & Therapeutics	.83				0.76
Musculoskeletal	.77		.31		0.70
Neuroscience	.86				0.76
Family Case Study				.94	0.96
Oncology Case Study		.94			0.89
OSCE exam	.44		.78		0.80

Footnote: Note that Factor loadings <0.3 are suppressed.

These conclusions were confirmed by a non-parametric PCA. In the loading plot the main entries matched those of the standard PCA, but there were superficial changes in some of the weak correlations, none of which challenged the general interpretation of the model.

### The relationship between selection to assessment factors

The decision to split the data into two groups, Selection and Assessment, before applying factor analysis was taken to allow us to simplify the data structure. Once we had succeeded in reducing the dimensionality of the data set to a more manageable level we could consider the relationship between the two groups. As a result of using orthogonal rotations, the factor model for each group is internally orthogonal. That is to say that within a model all factors would be uncorrelated with each other. We therefore considered all inter-model factor correlations, in which one factor was taken from each model. This would tell us whether any of the 3 main ‘components’ of selection (Academic record, GAMSAT score, interview) would have been useful indicators of how that student went on to perform in the main 4 ‘components’ of assessment (General Ability, Oncology, OSCE, Family Case Study).

The full set of cross-model correlations is given in Table 9. Only one relationship is at all significant, and this is between the student's previous academic record and their general exam performance. A limited number of significant correlations in itself is not that surprising, and can often be attributed to small sample size. What is striking here is the stark contrast between one very strong correlation and the other pair-wise comparisons, which show no evidence of any relationship at all.

**Table 9:** The correlation structure between the ‘Selection Factors’ (factors 1 to 3) and the ‘Assessment Factors’ (4 to 7).

	Factor 1: ‘Academic Record’	Factor 2: ‘GAMSAT’	Factor 3: ‘Interview’
Factor 4: ‘General Ability’	0.55**	0.11	−0.06
Factor 5: ‘Oncology’	−0.15	0.10	0.09
Factor 6: ‘OSCE’	−0.12	−0.15	0.05
Factor 7: ‘Family Case Study’	0.02	0.05	0.10

There was an expectation that there would be a clear distinction between different groups of modules, based either on their content or method of assessment. What we observed was that there was little evidence for grouping based on content and quite strong evidence for grouping by assessment type. There was an exceptionally high degree of correlation between members of the General Ability Assessment factor group (Alimentary through to Neuroscience), giving a high measure of internal consistency (Cronbach's α = 0.926).

It was expected that previous academic record would provide a good indicator of performance and this was borne out by the analysis. In fact it is the only good indicator we were able to identify. The GAMSAT exams are intended to provide a more specific measure of the suitability of a student for the study of medicine than prior academic record, but this is only partially supported by our findings. On the one hand it appears that they provide more information than is contained simply in a student's academic record, shown by its dominance of the second factor in the analysis of the selection data, but this additional information does not correlate with any element of the measures currently used to assess students. Given that GAMSAT 2 focuses primarily on written communication it seems likely that the GAMSAT exams are generally more demanding in this respect and this may form the basis of the GAMSAT factor. It is possible (although by no means obvious) that this link with written communication skills could provide some insight into the oddly strong correlation between Factor 2 and Age. That the interview process provides so little insight into performance is curious, as motivation and communication are two of the key themes on which students are assessed. The obvious explanation for this is that the skills and aptitudes identified in interview are real but not tested independently during assessment, but it could also suggest that the interview process is either invalid or unreliable.

It is worth noting though, that the suggestion we have made based on the data that the method of assessment is more important than the subject is somewhat contradicted by the low correlation between the Oncology and Family Case Studies (ρ = −0.05). Given that both of these are conducted and assessed in a broadly similar fashion we may have expected a closer agreement in the marks awarded to an individual student although there are, in practice, many differences between the modules. In particular, during family case studies, students work in pairs while in Oncology case studies they work alone, although in both modules reports are submitted individually. It is also surprising that the skills required to perform in the case studies and the OSCE exam do not appear correlated with either Factor 2 (GAMSAT) or 3 (Interview), which should be testing aptitude, reasoning and communication skills.

To test the robustness of these findings we performed a non-linear canonical correlation with the two sets, the selection and assessment variables (see Table 10). While the heavy transformation of the data inherent in the method and the absence of any test of significance in this procedure makes interpretation difficult, the results generally support our original findings. It was found that five dimensions gave the clearest results, with several dimensions suggested in order of decreasing strength. The first three dimensions strongly support the link between prior exam results and assessment by examination during the course. The second dimension also gives some support to the negative relationship between interview performance and exam results (and the contrary nature of Oncology). The fourth dimension is difficult to interpret, but the fifth indicates the negative link between interview performance and the OSCE score. Overall, though, it is also quite clear that the loadings are dominated by those linking previous exam performance with success examination based modules, supporting the original PCA.

**Table 10:** The correlation structure between the ‘Selection’ and ‘Assessment’ variables identified through non-linear canonical correlation.

	Dimension 1	Dimension 2	Dimension 3	Dimension 4	Dimension 5
GCSE points	0.506	−0.33		−0.303	
A-level points	0.503		0.461	0.354	
GAMSAT 1	0.72				
GAMSAT 2					−0.527
GAMSAT 3	0.616				
Sum of Interview Marks		0.363			0.385
Age				−0.301	
Alimentary	0.402			−0.546	
Cardiovascular & Respiratory	0.424		0.322		
Development, Growth & Reproduction	0.504	−0.409			0.428
Health in Society	0.765		0.327		
Homeostasis					
Human Structure		−0.401		−0.378	
Infection and Immunity					
Mechanisms of Disease & Therapeutics	0.789				
Musculoskeletal	0.318	−0.434	0.408	−0.386	
Neuroscience	0.468			−0.469	
Family Case Study			0.369		
Oncology Case Study		0.381		−0.329	
OSCE exam				−0.463	0.468

Footnote: Note that Factor loadings <0.3 are suppressed.

## Discussion

At the outset our intention was to examine the way we perform assessments, both for prospective students undergoing selection but also in measuring their progress once they are enrolled. The testing regime is predicated on the assumption that there is a stable human characteristic that equates to a ‘good doctor’. The admissions process attempts to measure this characteristic and then selects the candidates that will make ‘good doctors’. The in-course assessments then measure the progress of each student, and fail any candidate who falls below the threshold of acceptable performance. As an outcome of this analysis we may be forced to reassess either this assumption, or our methods of assessment.

In an initial exploratory analysis we found little indication that the background of students resulted in differing performance, and even these marginal variations quickly disappeared over the duration of the teaching. This basic analysis was followed by applying factor analysis to both selection and internal assessment data. The aims of factor analysis can be considered analogous, but opposite, to the concept of the examination process in general. In any exam we try and assess a trait (such as effectiveness as a ‘good doctor’) based on a set of proxy measurements (the examinations). In factor analysis we can attempt to reverse-engineer the problem: are a large set of measurements the result of a smaller set of distinct, but unknown, underlying traits (factors)? Being in essence a purely statistical process, it is often difficult to assign meaning to the resulting factors, however here we believe that the data reveal strong traits that are easy to interpret.

We found robust patterns in both selection and assessment results, each able to represent the data with a small number of factors. The selection data was dominated by a factor of prior academic performance, which partially overlapped with GAMSAT scores. The ‘Interview’ factor varied independently of the other factors. The assessment data was also dominated by a general exam ability factor (most of the ‘academic’ subjects), while the few modules assessed by coursework or practical examination were identified as separate factors. These results suggest that we are identifying differing characteristics at the interview than those available simply from examination statistics. Similarly, during the course, we have assessments that measure a range of skills that are not necessarily correlated (such as general exam scores vs the performance in the Family Case Study). However, when we combine our identified factors, there is a striking lack of correlations between our selection and performance criteria. There was only a strong correlation between the measure of ability in examinations from each set of criteria. The lack of any other correlations, including any correlation with the Interview Factor or any correlation between the case studies/course work and selection criteria was notable, although the internal reliability of some selection processes is established [11]. Interview scores are therefore measuring different characteristics to academic ability, but these traits are not manifest in the wide range of assessment factors. We accept that the use of global scores such as the OSCE mark and interview score prevent us from working with the constituent parts of both of these constructs, and it is possible that some insight may have been lost as a result. In our case a breakdown of marks was not available, and in any case it seems likely that any effects identified would have been relatively small compared to the impact of General Ability.

As a whole, these data provide little support for the assumption that ‘good doctor’ traits can be reliably identified from simple selection criteria, or measured in academic performance. The lack of correlation between admissions scores and assessment scores indicate that these either measure independent variables or that the construct is not stable over time. The one stable construct appears to be academic ability, evident in the strong correlation between prior academic record and certain module marks and the high correlation within these modules. This suggests that multiple assessments of academic ability during a medical course have little utility as performance indicators, although the effect of assessment on learning is well recognised (both good and bad).

The lack of correlation with the other three in-course assessment factors (Oncology project, OSCE and Family Case Study) would suggest that they either measure different traits or that the tests are unreliable. Unfortunately the single mark assigned to the two projects (Oncology and Family Case Studies) precludes measures on internal reliability. The only way to distinguish between these possibilities would be by longitudinal analysis over several years. The lack of correlation between other areas of the admissions process and academic marks indicates that we should modify either our admissions process or assessment scheme so that we select students who are most likely to succeed. This is likely to involve the development of more sophisticated admissions tests that seek to measure the required constructs and which seek to predict linked in-course assessments. Another obvious area of investigation is whether it is possible to reliably measure the ‘professionalism’ of students either during the admissions process or during their time as students and whether this correlates with interview score.

Although the construct of a ‘good doctor’ may not be a reality, these data suggest we are not currently able to reliably measure any student characteristic apart from general academic ability, providing support for a reduction in the testing of general academic ability and the development/validation of assessments of more sophisticated assessment tools.
